# Multiple phenotype association tests based on sliced inverse regression

**DOI:** 10.1186/s12859-024-05731-8

**Published:** 2024-04-04

**Authors:** Wenyuan Sun, Kyongson Jon, Wensheng Zhu, the Alzheimer’s Disease Neuroimaging Initiative

**Affiliations:** 1 Key Laboratory for Applied Statistics of MOE, School of Mathematics and Statistics, Northeast Normal University, Changchun, 130024 Jilin China; 2 Department of Mathematics, College of Science, Yanbian University, Yanji, 133002 Jilin China; 3 School of Mathematical Sciences, Harbin Normal University, Harbin, 150025 Heilongjiang China; 4 Faculty of Mathematics, Kim Il Sung University, Pyongyan , 999093 Democratic People’s Republic of Korea

**Keywords:** Sliced inverse regression, Sufficient dimension reduction, Dimension reduction

## Abstract

**Background:**

Joint analysis of multiple phenotypes in studies of biological systems such as Genome-Wide Association Studies is critical to revealing the functional interactions between various traits and genetic variants, but growth of data in dimensionality has become a very challenging problem in the widespread use of joint analysis. To handle the excessiveness of variables, we consider the sliced inverse regression (SIR) method. Specifically, we propose a novel SIR-based association test that is robust and powerful in testing the association between multiple predictors and multiple outcomes.

**Results:**

We conduct simulation studies in both low- and high-dimensional settings with various numbers of Single-Nucleotide Polymorphisms and consider the correlation structure of traits. Simulation results show that the proposed method outperforms the existing methods. We also successfully apply our method to the genetic association study of ADNI dataset. Both the simulation studies and real data analysis show that the SIR-based association test is valid and achieves a higher efficiency compared with its competitors.

**Conclusion:**

Several scenarios with low- and high-dimensional responses and genotypes are considered in this paper. Our SIR-based method controls the estimated type I error at the pre-specified level $\alpha$.

## Introduction

In recent biomedical research, Genome-Wide Association Studies (GWAS) often requires the simultaneous consideration of multiple phenotypes. It has been shown that jointly analyzing the multiple phenotypes together can increase statistical power to detect genetic variants [1, 2]. Introducing more information through the joint analysis will benefit revealing the complex relationship that may be undiscovered by the single phenotype analysis [3]. Meanwhile, the progressive improvements in data collection techniques have made it possible to measure more types of high-dimensional data on the same subject.

So far, the common strategies used to detect genetic associations in the joint analysis of multiple phenotypes can be roughly classified into several categories, such as regression model-based methods, nonparametric association methods, and *p* value correction methods. The regression model-based methods mainly exploit multivariate regression models [4–6], generalized estimating equations (GEEs), and mixed effects models [7–9]. As functional regression models perform well in most cases, Chui et al. [10] extended them to meta-analysis of pleiotropy traits, and Wang et al. [11] developed multivariate functional linear models and hypothesis testing procedure to test the association between multiple quantitative traits and multiple genetic variants in one genetic region. As a representative of the nonparametric association method, Zhang et al. [12] tested any hybrid of dichotomous, ordinal, and quantitative traits based on a generalization of Kendall’s tau. Zhu et al. [13] extend their method to accommodate covariates and proposed a nonparametric covariate-adjusted association test. Among the representative *p* value correction methods, one approach is Fisher’s combined method [14], which integrated the results from standard univariate analysis *p* value, and has been extended to dependent univariate test. Another approach called the minimum of the *p* value (Minp) method [15] has been applied to independent test studies to improve power when a SNP affects only a very small number of multiple phenotypes, but it is less powerful for denser signals. Sluis et al. [16] proposed the TATES method, which has good power in the presence of very few association signals but can lose its dominance otherwise.

Nevertheless, these methods focus only on the low-dimensional or moderate numbers of phenotypes. To this end, several dimension reduction methods, including principal component (PC) analysis, have been developed to reduce the high dimensionality of the phenotypes. Liu and Li [2] proposed the PC-based tests to take the high dimensional phenotypes into account and proved how to combine PCs together to achieve better power. But in fact, the PC-based tests consider only a single SNP, which makes it impossible to directly extend these tests to study the association between high dimensional phenotypes and multiple genotypes. Actually, with the development of next-generation sequencing technologies, recent GWAS usually collects high-dimensional SNPs and phenotypes. The implementation of association study often encounters other difficulties associated with the extremely high computational burden.

As another attempt to cope with the excessiveness of variables, Cook [17] introduced the idea of sufficient dimension reduction (SDR), which assumes that the response variable relates to only a few linear combinations of the many covariates. In this paper, we intend to reduce the dimension of the multivariate phenotype $\varvec{y}$ without loss of information about the multiple genotypes $\varvec{g}$ based on the idea of SDR, where the dimension of $\varvec{y}$ could be large. To this end, we use the sliced inverse regression (SIR) proposed by Li [18] to seek the effective dimension-reduction (e.d.r) direction and propose a SIR-based testing method for genetic association with multiple phenotypes. Different from the principal component analysis, the motivation behind the SIR is to preserve regression information during carrying out dimension reduction of multivariate phenotype, so that the resulting variates capture important features of the regression relationship between the multivariate phenotypes and multiple phenotypes. The simulation studies illustrate that the type  I error rates of our proposed tests are well-controlled and that the power is robust and powerful. We also apply the proposed SIR-based test to a real dataset, the Alzheimer’s Disease Neuroimaging Initiative (ADNI) dataset, and successively identify the new genetic variants.

## Methods

### SIR-based association test of multiple phenotypes

#### Data structure and linear regression

Suppose that data are collected from a population-based sequencing study with *n* independent individuals. For each individual, we observe *q* disease phenotypes and genotypes at *k* SNPs. Let the phenotype vector and genotype vector be $\varvec{y}=(y_{1}, \dots , y_{q})^T$ and $\varvec{g}=(g_{1}, \dots , g_{k})^T$, respectively. Then, the observations of genotypes and trait measurements for *n* individuals are denoted as an $n\times k$ matrix ${\textbf{G}} = (\varvec{g}_{1},\dots , \varvec{g}_{n})^{T}$ and an $n\times q$ matrix ${\textbf{Y}} = (\varvec{y}_{1}, \dots , \varvec{y}_{n})^{T}$, respectively. Here, notations of $\varvec{g}_{i}$ and $\varvec{y}_{i}$ refer to the instances of $\varvec{g}$ and $\varvec{y}$ for *i*-th individual ($i=1,\dots ,n$). In this study, the *q* could be large, so detecting disease-associated genetic variants with large *q* is very challenging. In addition, the effects of the correlation between phenotypes and the direction of genetic effects should be considered. For brevity, we focus on the most popular continuous phenotypes only and consider a multivariate linear regression model with large *q*.

To describe the relationship between the phenotype $\varvec{y}$ and the genotype $\varvec{g}$, we propose the following linear model:

1 $$\begin{aligned} \varvec{y} ={\textbf{B}}^{T} \varvec{g} + \varvec{\varepsilon }, \end{aligned}$$

where ${\textbf{B}} = (\varvec{\beta }_{1}^T \dots , \varvec{\beta }_{k}^T)^{T}$ is a $k \times q$ matrix of the regression coefficients, $\varvec{\beta }_{j}=(\beta _{j1},\dots , \beta _{jq})$ is a *q*-dimensional row vector of regression coefficients for the *j*-th SNP, which represents the effects of the *j*-th SNP on *q* phenotypes, and $\varvec{\varepsilon }$ is the *q*-dimensional error vector with zero mean and true covariance matrix, which is often unknown. Here, the intercepts are omitted with $\varvec{y}$ being properly centered. We can rewrite the above model in the following matrix form:

2 $$\begin{aligned} {\textbf{Y}} = {\textbf{G}}{\textbf{B}}+ {\textbf{E}}, \end{aligned}$$

where ${\textbf{E}}$ = $(\varvec{\varepsilon }_{1}, \dots , \varvec{\varepsilon }_{n})^T$ is the $n \times q$ error matrix with $\varvec{\varepsilon }_{i}$ being the *q*-dimensional error vector for *i*-th individual.

Our primary interest lies in testing whether the genetic markers ${\varvec{g}}$ are associated with the traits ${\varvec{y}}$. To address this problem, two strategies are commonly considered. Firstly, testing the effects of the *j*-th SNP on *q* phenotypes is equivalent to testing the null hypothesis $H_{0}: \varvec{\beta }_j={\varvec{0}}$ against the alternative hypothesis $H_{1}$ that at least one element of $\varvec{\beta }_j$ is not equal to zero. In this case, the Wald-type statistic $T_1={\hat{\varvec{\beta }}}_j \left[ {\text {Cov}}({\hat{\varvec{\beta }}}_j)\right] ^{-1}{\hat{\varvec{\beta }}}_j^{T}$ is adopted, where ${\hat{\varvec{\beta }}}_j$ is the maximum likelihood estimator (MLE) of $\varvec{\beta }_j$ and ${\text {Cov}}({\hat{\varvec{\beta }}}_j)$ is its covariance matrix. The test statistic $T_1$ has *q* degrees of freedom. When *q* is large and heterogeneous effects exist, especially when a variant only affects a subset of traits, the test statistic may be less powerful due to the large degrees of freedom. Furthermore, conducting the association study with multiple tests often results in a significant loss of statistical power due to a large number of comparisons. The second strategy involves considering the association between all *k* SNPs and *q* phenotoypes, which is equivalent to testing the null hypothesis $H_{0}: {\textbf{B}}_1={\varvec{0}}$. The Wald-type statistic can be rewritten as $T_2=\hat{{\textbf{B}}}_1 \left[ {\text {Cov}}(\hat{{\textbf{B}}}_1)\right] ^{-1}\hat{{\textbf{B}}}_1^{T}$, where $\hat{{\textbf{B}}}_1$ is the MLE of ${\textbf{B}}_1=(\beta _{11}, \dots , \beta _{1q}, \beta _{21}, \dots , \beta _{2q}, \dots , \beta _{k1}, \dots , \beta _{kq})$. The test statistic $T_2$ has *kq* degrees of freedom and the implementation of the association study with high-dimensional phenotypes often encounters other difficulties concerning the extremely high computational burden. Since both $T_1$ and $T_2$ have their own disadvantages for large degrees of freedom, a common solution is to reduce the dimensionality of responses and/or predictors. In the following subsections, we present a SIR-based dimension reduction of $\varvec{y}$ and test procedures with reduced phenotypes ${\varvec{y}}$.

#### SIR-based dimension reduction of ${{\varvec{y}}}$

As the dimension of phenotype $\varvec{y}$ is very large, it is highly desirable that interesting features of high-dimensional data are retrievable from low-dimensional projections. PCA is perhaps the most well-known method for reducing dimensionality. But since the procedure is carried out without using the predictor variable, certain interesting regression variables may be lost in the reduction process and hardly capture important features of the regression relationship between response variables and predictors. Another attempt to cope with the excessiveness of variables is the SDR approach, which assumes that only a few linear combinations of original variables are sufficient to reveal the information within them without changing their explanatory effect on the response variable. Identifying these linear combinations is the goal of dimension reduction. To this end, many authors have utilized the so-called sliced inverse regression (SIR) method proposed by [18], which focuses on the inverse regression method by reversing the relation between the response and predictor variables to benefit from the response variable being, usually, of lower dimension than predictor vector. Here, we adopt the SIR method to reduce the dimension of the multivariate response ${\varvec{y}}$ without loss of information about the multiple genotypes ${\varvec{g}}$.

To better understand the SIR method, we consider the following forward regression model:

3 $$\begin{aligned} \varvec{g}=f({\varvec{S}}_1^T\varvec{y}, {\varvec{S}}_2^T\varvec{y}, \dots , {\varvec{S}}_d^T\varvec{y}, \varvec{\epsilon }), \end{aligned}$$

where ${\varvec{S}}_1, \dots , {\varvec{S}}_d$ are unknown e.d.r directions, *d* is the number of dimensions one want to achieve, $\varvec{\epsilon }$ is independent of $\varvec{y}$, and *f* is an arbitrary unknown function on $\mathbb {R}^{d+1}$. When the model (3) holds, the *q*-dimensional $\varvec{y}$ can be projected onto the *d*-dimensional subspace with $d \ll q$, so that interesting features of the high-dimensional $\varvec{y}$ are compressed by low-dimensional projections. If $\varvec{g}$ is statistically independent of $\varvec{y}$ when ${\varvec{S}}_m^T\varvec{y}, m=1,\dots ,d$, are given, it is sufficient to focus only on the *d* reduced variables $\varvec{S}_m^T\varvec{y}$’s for studying the relationship between $\varvec{g}$ and $\varvec{y}$. At this point, the column space of a $q\times d$ matrix ${\textbf{S}}=\left( {\varvec{S}}_1, \dots , {\varvec{S}}_d\right)$ becomes a SDR subspace.

To reduce the dimension as much as possible, we are often interested in the SDR subspace with the smallest dimension. Under mild conditions [17, 19], the intersection of all SDR subspaces is still an SDR subspace, and the smallest SDR subspace is called the central subspace. For notational simplicity, in the following, we assume the central subspace to be estimated is spanned by a $q\times d_{0}$ basis matrix, denoted by ${\textbf{S}}_0=\left( {\varvec{S}}_1, \dots , {\varvec{S}}_{d_0}\right)$. If we further assume that $\varvec{y}$ has been standardized to ${\varvec{z}}$, under a linearity condition that $E(\varvec{z}\mid {\textbf{S}}_0^T\varvec{z})$ is linear in ${\textbf{S}}_0^T\varvec{z}$, it is guaranteed that the $E(\varvec{z}\mid \varvec{g})$ belong to the space spaned by $\varvec{S}_1, \dots , \varvec{S}_{d_0}$ [18, 20]. Then, we can estimate the central subspace by applying a principal component analysis to the random vector $E(\varvec{z}\mid \varvec{g})$, following the approach proposed by [18]. Equivalently, we can derive a basis of central subspace by solving

4 $$\begin{aligned} \mathop {\arg \max }\limits _{{\textbf{S}}_0^T{\textbf{S}}_0={\textbf{I}}_{d_0}}\textrm{tr}\left( {\textbf{S}}_0^T{\text {Cov}}\left[ E(\varvec{z} \mid \varvec{g})\right] {\textbf{S}}_0\right) , \end{aligned}$$

the solution of which is formed by the $d_0$ leading eigenvectors of ${\text {Cov}}\left[ E\left( \varvec{z} \mid \varvec{g}\right) \right]$, where ${\text {Cov}}\left[ E\left( \varvec{z} \mid \varvec{g}\right) \right]$=$E\left[ E\left( \varvec{z}\mid \varvec{g}\right) E\left( \varvec{z}\mid \varvec{g}\right) ^T\right]$ and $\textrm{tr}\left( \cdot \right)$ represents the sum of the eigenvalues of the matrix ${\text {Cov}}[ E(\varvec{z} \mid \varvec{g})]$.

In this study, our concern is focused on testing the association between marker genes and multiple traits. However, under the null hypothesis, the traits and genes are independent. In this case, ${\text {Cov}}[ E(\varvec{z} \mid \varvec{g})]= E\left[ \left( E\left( \varvec{z}\mid \varvec{g}\right) - E\left[ E\left( \varvec{z}\mid \varvec{g}\right) \right] \right) \left( E\left( \varvec{z}\mid \varvec{g}\right) - E\left[ E\left( \varvec{z}\mid \varvec{g}\right) \right] \right) ^{T}\right] =0$, and the estimation of ${\text {Cov}}\left[ E(\varvec{z} \mid \varvec{g})\right]$ will be very small and close to 0 in actual situations, then it becomes challenging to directly apply the PCA on the ${\text {Cov}}\left[ E(\varvec{z} \mid \varvec{g})\right]$ by following the SIR method suggested by [18]. Fortunately, we see the relation between ${\text {Cov}}\left[ E\left( \varvec{z} \mid \varvec{g}\right) \right]$ and $E[{\text {Cov}}(\varvec{z} \mid \varvec{g})]$ as

5 $$\begin{aligned} E\left[ {\text {Cov}}(\varvec{z} \mid \varvec{g})\right] ={\text {Cov}} (\varvec{z})-{\text {Cov}}\left[ E(\varvec{z} \mid \varvec{g})\right] =I-{\text {Cov}}\left[ E(\varvec{z} \mid \varvec{g})\right] . \end{aligned}$$

Alternatively, by applying the eigenvalue decomposition of $E[{\text {Cov}}(\varvec{z} \mid \varvec{g})]$, we can determine the standardized e.d.r. directions which are the eigenvectors associated with the $d_0$ smallest eigenvalues. This procedure equivalently derives a basis of central subspace by solving

6 $$\begin{aligned} \mathop {\arg \min }\limits _{{\textbf{S}}_0^T{\textbf{S}}_0={\textbf{I}}_{d_0}}\textrm{tr}\left( {\textbf{S}}_0^T E\left[ {\text {Cov}}(\varvec{z} \mid \varvec{g})\right] {\textbf{S}}_0\right) , \end{aligned}$$

the solution of which is formed by the $d_0$ leading eigenvectors of $E\left[ {\text {Cov}}(\varvec{z} \mid \varvec{g})\right]$.

In the following, we give a detailed estimation procedure utilizing the SIR scheme based on the observed data ($\varvec{g}_{i},\varvec{y}_{i}$), $i = 1, \dots , n$:

- Standardize $\varvec{y}_i$ by an affine transformation to get $\varvec{z}_i={\hat{\Sigma }}_{{\varvec{y y}}}^{-1 / 2}(\varvec{y_{i}}-\overline{\varvec{y}})$, ($i = 1, \dots , n$), where ${\hat{\Sigma }}_{\varvec{y y}}$ and $\overline{\varvec{y}}$ are the sample covariance matrix and sample mean of $\varvec{y}_1,\dots , \varvec{y}_n$, respectively.
- Divide the range of $\varvec{g}$ into the *H* slices, $I_{1},\dots ,I_{H}$; let the proportion of the $\varvec{g}_{i}$’s falling into the *h*-th slice be ${\hat{p}}_{h}$ ($h=1, \dots , H$), that is, ${\hat{p}}_{h}$=$(1 / n) \sum _{i=1}^{n} \delta _{h}\left( \varvec{g}_{i}\right)$, where $\delta _{h}\left( \varvec{g}_{i}\right)$ takes the value 0 or 1 depending on whether $\varvec{g}_{i}$ falls into the *h*-th slice $I_{h}$ or not.
- Within each slice, compute the sample covariance of the $\varvec{z}_{i}$’s, denoted by $\hat{\varvec{v}}_h~(h=1, \dots , H)$, that is, $\hat{\varvec{v}}_h=\left( 1 / n {\hat{p}}_{h}\right) \sum _{\varvec{g}_{i} \in I_{h}} \varvec{z}_{i}\varvec{z}_{i}^T$.
- Conduct a (weighted) principal component analysis for the data $\hat{\varvec{v}}_h~ (h=1, \dots , H)$: firstly, form the weighted mean value $\hat{{\textbf{E}}}=\sum _{h=1}^{H} {\hat{p}}_{h} \hat{\varvec{v}}_h$; next, find the eigenvalues and the eigenvectors for $\hat{{\textbf{E}}}$.
- Let $\hat{\varvec{\eta }}_{m}~(m=1,\dots , d_0)$ be the *m* smallest eigenvectors. By transforming back to the original scale, output ${\hat{{\varvec{S}}}}_{m}=\hat{\varvec{\eta }}_{m} {\hat{\Sigma }}_{\varvec{yy}}^{-1 / 2}~(m=1,\dots , d_0)$ which are in the e.d.r. space.

When dividing the range of $\varvec{g}$, the most natural choice is to divide it into $H=3^k$ slices, considering the fact that each locus of the *k* SNPs takes values in $\{0, 1, 2\}$. However, if the dimension of genotypes is very high, then such a straightforward implementation, while theoretically possible, is intractable in practice. This is because there will be many empty slices due to a massive number of slices and the limited sample size, making it impossible to calculate the covariance in those empty slices. For this reason, we adopt an alternative way of dividing the range of $\varvec{g}$ and grouping individuals, following the approach mentioned in [21]. Specifically, we first estimate the genetic relatedness matrix to measure genetic similarity among individuals and divide the range of $\varvec{g}$ in terms of that similarity. Next, we merge adjacent slices so that the number of individuals in each slice is not less than 5. Then, we calculate the conditional covariance of each slice according to the estimation procedure for $E[{\text {Cov}}(\varvec{z} \mid \varvec{g})]$, which is described above.

### SIR-based association test with reduced phenotypes

After estimating the $d_0$ standardized e.d.r directions, the *q*-dimensional $\varvec{y}$ can be projected onto the $d_0$-dimensional central subspace with $d_0 \ll q$. Then, the predictor variable $\varvec{g}$ is related to only $d_0$ linear combinations, $\varvec{S}_1^T\varvec{y},\dots ,\varvec{S}_{d_0}^T\varvec{y}$, and it is sufficient to focus only on them. According to [17, 18], it is fair to say that one-component model ($d_0=1$) has prevailed, therefore, for the sake of simplicity, only case of $d_0=1$ is considered in this paper.

Consequently, the large dimensional phenotype $\varvec{y}_i~(i=1,\dots ,n)$ can be transformed into $\tilde{{y}}_{i}\in \mathbb {R}$ without loss of information on the corresponding genotype $\varvec{g}_i$. At this point, we can investigate the relationship between phenotype $\varvec{y}$ and genotype $\varvec{g}$ in the following form:

7 $$\begin{aligned} {\tilde{{\varvec{Y}}}}= {\textbf{G}}{\tilde{\varvec{\beta }}} + {\tilde{{\varvec{E}}}}, \end{aligned}$$

where ${\tilde{{\varvec{E}}}}$ = $({\tilde{\varvec{\varepsilon }}}_{1}, \dots , {\tilde{\varvec{\varepsilon }}}_{n})^T$ is an $n \times 1$ error vector with ${\tilde{\varvec{\varepsilon }}}_{i}$ being the error term for *i*-th individual, ${\tilde{{\varvec{Y}}}} = (\tilde{{y}}_{1}, \dots , \tilde{{y}}_{n})^{T}$ is an $n\times 1$ vector of the traits, ${\tilde{\varvec{\beta }}}$ is a regression coefficient vector.

We aim to test whether the set of genetic markers is associated with phenotype after dimension reduction. This is equivalent to testing the null hypothesis $H_{0}: {\tilde{\varvec{\beta }}}={\textbf{0}}$ against the alternative hypothesis $H_{1}$ that at least one element of ${\tilde{\varvec{\beta }}}$ is not equal to zero. In this case, Wald-type statistic $\tilde{T}=\left( \widehat{{\tilde{\varvec{\beta }}}}\right) ^{T} \left[ {\text {Cov}}(\widehat{{\tilde{\varvec{\beta }}}})\right] ^{-1}\left( \widehat{{\tilde{\varvec{\beta }}}}\right)$ no longer follows the chi-square distribution under the null hypothesis, where $\widehat{{\tilde{\varvec{\beta }}}}$ is the MLE of ${\tilde{\varvec{\beta }}}$ and ${\text {Cov}}(\widehat{{\tilde{\varvec{\beta }}}})$ is its covariance matrix. We use a permutation procedure to establish the null distribution of $\tilde{T}$. The permutation is done by randomly assigning the genotypes while keeping the phenotypes for each individual. For each permuted data set, we use (7) to calculate $\tilde{T}$ as we have done by using the original data set. We repeat this procedure 1000 times to generate the distribution of $\tilde{T}$ under the null hypothesis of no association between multiple genotypes and the phenotypes. This testing strategy, in the sense that it is about all coefficients, can be seen as a global test.

In addition to this, it is also possible to focus on the association of the single SNP after dimension reduction. To this end, we apply Bonferroni correction to adjust for multiple testing involving *k* markers, which is equivalent to testing the null hypothesis $H_{0}: \tilde{\beta }_k={\textbf{0}}$ against $H_{a}: {\tilde{\beta }}_k\ne {\textbf{0}}$. The model for this case is

8 $$\begin{aligned} {\tilde{y}}_i= g_{ik}{\tilde{\beta }}_k + {\tilde{\varvec{\varepsilon }}}_{i},~~i=1,\dots , n, \end{aligned}$$

where ${\tilde{\beta }}_k$ is a regression coefficient and ${\tilde{\varvec{\varepsilon }}}_{i}$ is an error term for *i*-th individual. The test statistic $\tilde{T}_k^2=\hat{{\tilde{\beta }}}_{k}^2/{\text {Var}}\left( \hat{{\tilde{\beta }}}_k\right)$ also does not follow the chi-square distribution under the null hypothesis, where $\hat{{\tilde{\beta }}}_k$ is the MLE of ${\tilde{\beta }}_k$ and ${\text {Var}}(\hat{{\tilde{\beta }}}_k)$ is its variance. To carry out the association test, we apply the permutation procedure to estimate the distribution of $\tilde{T}_k$.

## Simulation studies

We conduct a series of simulation studies to evaluate the numerical performance of the proposed association tests in comparison with eight other PC-based competing tests, such as PCA1, PCFisher, PCMinp, PCLC, Wald, WI, VC, and PCAQ [2]. In these PC-based tests, the PCA1 indicates using only the first principal component, the PCFisher can be viewed as a nonlinear combination of the PC *p* values, the PCMinp uses the minimum PC *p* value as a testing statistic, and other tests aim at constructing the linear or quadratic combinations of PCs weighted by the functions of eigenvalues.

We simulate the genotype $\varvec{g}_{i}=(g_{i1}, \dots , g_{ik})^T$ for the *i*-th individual at *k* SNPs, where the genotype of each SNP is sampled from a uniform distribution with a minor allele frequency (MAF=*p*) between 0.3 and 0.5 under the assumption of Hardy-Weinberg equilibrium. That is, $p_{aa}=(1-p)^{2}$, $p_{Aa} = 2p(1-p)$, and $p_{AA} =p^{2}$. The *q*-dimensional phenotype $\varvec{y}_{i}$ of the *i*-th individual is generated from the model (1), where $\varvec{\varepsilon }_i$ follows $N({\textbf{0}},\Sigma )$ with $\Sigma _{lm}=\rho ^{|l-m|}$ for $1\le l, m\le q$, and $\rho$ is the correlation coefficient between phenotypes. Note that the simulated data under the null hypothesis of $\varvec{\beta }={\textbf{0}}$ can be used to calculate type I errors, whereas the data under the alternative hypothesis saying that $\varvec{\beta }$ contains at least one nonzero element can be used to calculate powers for each method. Hereafter, this global test mentioned above is expressed as SIR in this study. Alternatively, based on the model (8), we can also perform the association test for each SNP separately, and adjust the test for all the SNPs through multiple testing procedure, named SIR-S.

In the simulation studies, we consider three scenarios: Scenario 1 is for the low-dimensional phenotype (*q* = 5 and 10) and low-dimensional genotype (*k* = 5 and 10); Scenario 2 is for the high-dimensional phenotype (*q* = 50 and 100) but low-dimensional genotype (*k* = 10); Scenario 3 is for both high-dimensional phenotype (*q* = 50 and 100) and genotype (*k* = 40 and 100). We set the nominal level of significance $\alpha =0.05$. Since the PC-based methods focus on the association test of single marker, here we apply Bonferroni correction to adjust for multiple testing involving *k* markers. In each scenario, we increase the correlation coefficient of phenotype in a series of $\rho$ = 0, 0.2, 0.5, 0.7. For each scenario, we generate 100,000 and 1000 simulated data sets for type I error evaluation and for power calculation, respectively.

### Scenario 1: low-dimensional phenotype and low-dimensional genotype

In this scenario, the dimension of phenotype is set to be *q* = 5 and 10, and the number of SNPs is set to be *k* = 5 and 10. We compare the power of each method in terms of the signal direction, signal strength, and the correlation structure among phenotypes. To this end, we consider different values of effect vector for each phenotype, specifically four cases for this scenario: Case 1 is for $k=5$, $q=5$; Case 2 is for $k=5$, $q=10$; Case 3 is for $k=10$, $q=5$; Case 4 is for $k=10$, $q=10$. Here, we let most $\varvec{\beta }_j$’s be zeros except for $\varvec{\beta }_{3}$ and $\varvec{\beta }_{4}$ being nonzeros. The effect vector of the third SNP $\varvec{\beta }_{3}$ on each phenotype is positive, except for Case 4, where its direction is mixed. The value of $\varvec{\beta }_{4}$ is given such that the fourth SNP is only associated with the second trait in all settings. The detailed setting of effect vectors is shown in Table 1.

**Table 1:** Low-dimensional setting of effect vectors in Scenario 1

Case 1	*k*=5, *q*=5	$\varvec{\beta }_{3}=(1.10,1.10,1.10,1.10,1.10)$
		$$\varvec{\beta }_{4}=(0.00,0.02,0.00,0.00,0.00)$$
Case 2	*k*=5, *q*=10	$\varvec{\beta }_{3}=(1.10,1.10,1.10,1.10,1.10,0.00,0.00,0.00,0.00,0.00)$
		$$\varvec{\beta }_{4}=(0.00,0.02,0.00,0.00,0.00,0.00,0.00,0.00,0.00,0.00)$$
Case 3	*k*=10, *q*=5	$\varvec{\beta }_{3}=(1.10,1.10,1.10,1.10,1.10)$
		$$\varvec{\beta }_{4}=(0.00,0.02,0.00,0.00,0.00)$$
Case 4	*k*=10, *q*=10	$\varvec{\beta }_{3}=(1.10,-1.10,1.10,-1.10,1.10,0.00,0.00,0.00,0.00,0.00)$
		$$\varvec{\beta }_{4}=(0.00,0.02,0.00,0.00,0.00,0.00,0.00,0.00,0.00,0.00)$$

$*$ The default value of other effect vectors ${\varvec{\beta }_j}$’s are ${\textbf{0}}$

**Table 2:** Empirical type I errors based on 100,000 replicates in Scenario 1

*n*	*k*	*q*	$\rho$	SIR	SIR-S	PCA1	PCFisher	PCMinp	PCLC	Wald	WI	$\textrm{VC}$	$\textrm{PCAQ}$
1000	5	5	0	0.05133	0.05156	0.04981	0.04669	0.05011	0.04989	0.04890	0.04766	0.04887	0.04709
			0.2	0.05284	0.05011	0.04824	0.04807	0.04078	0.04882	0.04800	0.04888	0.04740	0.04902
			0.5	0.05367	0.04898	0.04856	0.04818	0.04219	0.04923	0.04891	0.04954	0.04788	0.04980
			0.7	0.05105	0.04920	0.05109	0.04854	0.04679	0.04997	0.04967	0.05144	0.04962	0.05066
	5	10	0	0.05518	0.04927	0.04860	0.04966	0.05478	0.04841	0.04978	0.04988	0.04982	0.04974
			0.2	0.05372	0.05145	0.04908	0.05701	0.04920	0.04908	0.04962	0.04935	0.05015	0.05016
			0.5	0.04887	0.05219	0.05491	0.05031	0.04958	0.05088	0.04895	0.05011	0.05055	0.05067
			0.7	0.05250	0.05296	0.04897	0.05028	0.04835	0.05013	0.04882	0.04984	0.04975	0.04578
	10	5	0	0.05197	0.05709	0.05043	0.04851	0.04846	0.04835	0.04965	0.04906	0.04904	0.05030
			0.2	0.05065	0.05202	0.04998	0.05056	0.04889	0.05468	0.05103	0.04923	0.05020	0.05156
			0.5	0.05091	0.05305	0.04991	0.05024	0.04856	0.04963	0.05203	0.04785	0.04912	0.04947
			0.7	0.05026	0.05296	0.05016	0.04975	0.04383	0.04882	0.04816	0.04983	0.04237	0.04637
	10	10	0	0.05463	0.05097	0.49231	0.04812	0.04976	0.04975	0.04943	0.04841	0.04458	0.04947
			0.2	0.04974	0.04869	0.05021	0.04854	0.05004	0.05002	0.04955	0.04685	0.04857	0.04857
			0.5	0.05122	0.05124	0.05225	0.04874	0.04954	0.04966	0.04715	0.04793	0.04886	0.04789
			0.7	0.05098	0.05014	0.04847	0.04994	0.04759	0.05207	0.04864	0.05305	0.04980	0.04262
2000	5	5	0	0.05035	0.05193	0.05109	0.04988	0.04927	0.05023	0.04987	0.04699	0.04788	0.04998
			0.2	0.05254	0.05025	0.05024	0.04932	0.04366	0.04982	0.04769	0.04708	0.04903	0.04078
			0.5	0.05173	0.05097	0.05204	0.04872	0.04018	0.04897	0.04829	0.04908	0.04874	0.05003
			0.7	0.05018	0.05164	0.05208	0.04996	0.04579	0.04760	0.04887	0.05035	0.04878	0.04978
	5	10	0	0.05087	0.05597	0.04845	0.05701	0.04920	0.04877	0.04950	0.04981	0.04978	0.04788
			0.2	0.04975	0.05348	0.04831	0.05136	0.04798	0.05004	0.04698	0.04983	0.04827	0.04516
			0.5	0.05429	0.05607	0.04985	0.04879	0.04876	0.05190	0.04623	0.04637	0.04983	0.04792
			0.7	0.05052	0.05375	0.04812	0.04941	0.04791	0.05033	0.04682	0.04784	0.04813	0.04507
	10	5	0	0.05308	0.05469	0.05039	0.04793	0.04985	0.04955	0.04957	0.04875	0.04775	0.04778
			0.2	0.05246	0.05325	0.04831	0.04839	0.04947	0.04976	0.05012	0.04975	0.04337	0.04642
			0.5	0.05108	0.05198	0.05017	0.04884	0.04761	0.04383	0.04858	0.04953	0.04435	0.04936
			0.7	0.05073	0.05206	0.04994	0.04965	0.04879	0.04898	0.04838	0.04962	0.04332	0.04642
	10	10	0	0.05180	0.05249	0.04972	0.05089	0.05018	0.04886	0.04954	0.04874	0.04648	0.05260
			0.2	0.05455	0.05167	0.04886	0.04787	0.04956	0.04632	0.04989	0.04847	0.04887	0.05105
			0.5	0.05223	0.05228	0.04975	0.04897	0.04587	0.04958	0.05003	0.04996	0.04989	0.04988
			0.7	0.05042	0.05175	0.04847	0.04434	0.04989	0.05411	0.04885	0.05305	0.04880	0.05062

From the results summarized in Table 2, it is apparent to see that the estimated type I error values of both SIR and SIR-S methods for different values of *q*, *k*, and $\rho$ are very close to the true error level of $\alpha =0.05$ and the two methods have the well-controlled empirical type I error rates in most cases. For further comparisons, we also make the PC-based tests as additional baseline methods. Table 2 clearly shows that all the PC-based methods retain the empirical type I errors very well at the significance level in most cases. Notice that the type I error rate of the VC method has slightly conservative with the empirical type I error of 0.04237 when we set $k=10$ and $q=5$. Overall, the SIR and SIR-S methods can accurately control the empirical type I errors at the nominal level.

**Fig. 1:**
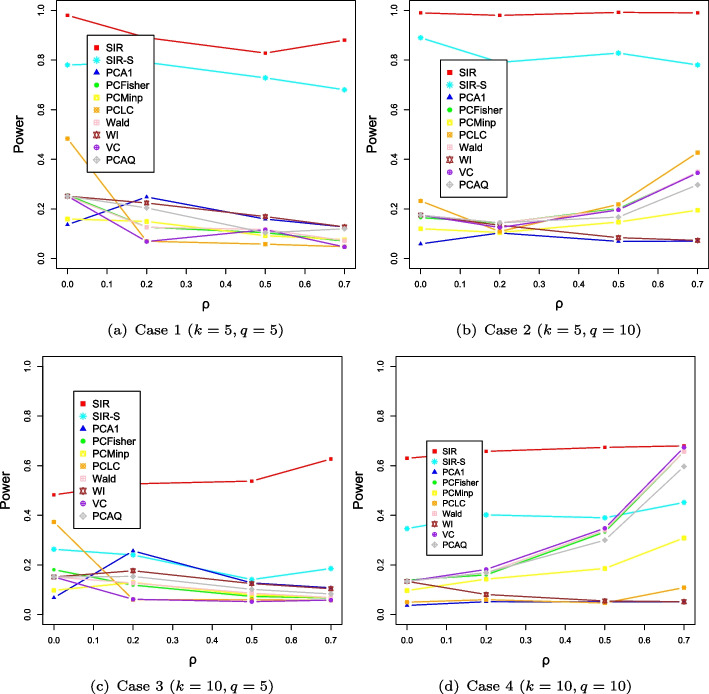
The evolution of power along with the varying correlation $\rho$ in the case of $n=1000$

**Fig. 2:**
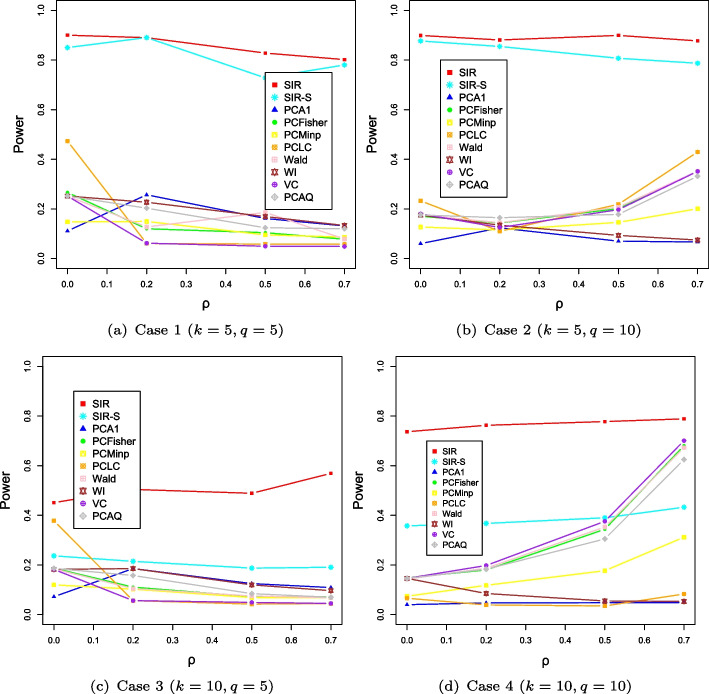
The evolution of power along with the varying correlation $\rho$ in case of $n=2000$

We further compare the empirical powers of the proposed tests with the existing PC-based methods. For each setup, we generate $n=1000$ and 2000 samples. The powers are calculated by the proportion of *p* values less than the significance level. We take the signal direction, signal strength, and the correlation structure among traits into account. Figures 1 and 2 show the powers of the ten comparative methods for different settings. We can see that the powers of the SIR and SIR-S methods are close to 1 and other PC-based methods are more powerless than the two methods in the case of $k=5$. In a nutshell, with the same number of genotypes, if the dimension *q* is equal to 5, the powers of PC-based methods will decrease as the correlation coefficient increases, but if the dimension *q* is equal to 10, the power increases contrarily. However, the proposed methods still have much higher power than the other alternative methods. Different from the case of $k=5$, we can see that the powers of the SIR and SIR-S methods decrease as the dimension of genotype *k* increases. The PCFisher, Wald, and VC have comparable performances to the proposed tests when the effect vectors are in a mixed direction for a strong phenotypic correlation. From Figs. 1 and 2, we know that both the SIR and SIR-S methods are sensitive to the direction of the signal. The increase in sample size has little effect on the power of all methods.

### Scenario 2: high-dimensional phenotype and low-dimensional genotype

To further show the performance of the proposed methods in the case of high-dimensional phenotype, we carry out additional simulations to compare our SIR and SIR-S methods with the other eight methods. Since Scenario 1 shows the sample size has little effect on the power for all methods, in this simulation, we only generate *n*=1000 individuals with different correlation structures of traits. The datasets are generated similarly to Scenario 1 except for the effect vectors. We consider three cases for this scenario: Case 1 is for $k=10$, $q=50$; Case 2 is for $k=10$, $q=50$; Case 3 is for $k=10$, $q=100$. The effect vector of the third SNP $\varvec{\beta }_{3}$ on the first five phenotypes is positive in Case 1 and Case 3, while the effect vectors in Case 2 have mixed directions. The setting of the different effect vectors is shown in Table 3. Table 4 summarizes the empirical type I errors of these methods for the association analysis. It is clear that all methods can control the empirical type I error well in most cases. Then, we compare the powers of our methods with the PC-based methods. When $q=50$, we have two cases and set the effects of the third SNP on the first five traits to be positive and mixed directions, respectively. In all cases, the fourth SNP is only associated with the second trait.

**Table 3:** The setting of effect vectors in Scenario 2

Case 1	$k=10,q=50$	$\varvec{\beta }_{3}=c(1.10,1.10,1.10,1.10,1.10,0.00,0.00,\dots ,0.00)$
		$$\varvec{\beta }_{4}=c(0.00,0.02,0.00,0.00,\dots ,0.00)$$
Case 2	$k=10,q=50$	$\varvec{\beta }_{3}=c(1.10,-1.10,1.10,-1.10,1.10,0.00,0.00,\dots ,0.00)$
		$$\varvec{\beta }_{4}=c(0.00,0.02,0.00,0.00,\dots ,0.00)$$
Case 3	$k=10,q=100$	$\varvec{\beta }_{3}=c(1.10,1.10,1.10,1.10,1.10,0.00,0.00,\dots ,0.00)$
		$$\varvec{\beta }_{4}=c(0.00,0.02,0.00,0.00,\dots ,0.00)$$

$*$ The default value of other effect vectors ${\varvec{\beta }_j}$’s are ${\textbf{0}}$

**Table 4:** Empirical type I errors based on 100,000 replicates in Scenario 2

*k*	*q*	$\rho$	SIR	SIR-S	PCA1	PCFisher	PCMinp	PCLC	Wald	WI	VC	PCAQ
10	50	0.0	0.05215	0.05168	0.04962	0.05175	0.05183	0.05304	0.05054	0.05344	0.05214	0.05038
		0.2	0.05167	0.05267	0.04782	0.04539	0.05264	0.05016	0.05163	0.04984	0.04993	0.05087
		0.5	0.05102	0.05196	0.04935	0.05023	0.04863	0.05127	0.04846	0.04887	0.05062	0.05090
		0.7	0.04985	0.05227	0.04935	0.04768	0.04996	0.05023	0.04963	0.04778	0.05123	0.05176
10	100	0.0	0.05015	0.05167	0.04916	0.05224	0.04985	0.04843	0.04908	0.05239	0.05105	0.05137
		0.2	0.05103	0.05070	0.05262	0.05143	0.04998	0.04765	0.05325	0.05008	0.04964	0.05661
		0.5	0.05234	0.05165	0.05305	0.04816	0.04623	0.04332	0.05233	0.05173	0.05141	0.05122
		0.7	0.05004	0.05296	0.05203	0.04984	0.04843	0.04793	0.04878	0.05013	0.05127	0.05027

**Fig. 3:**
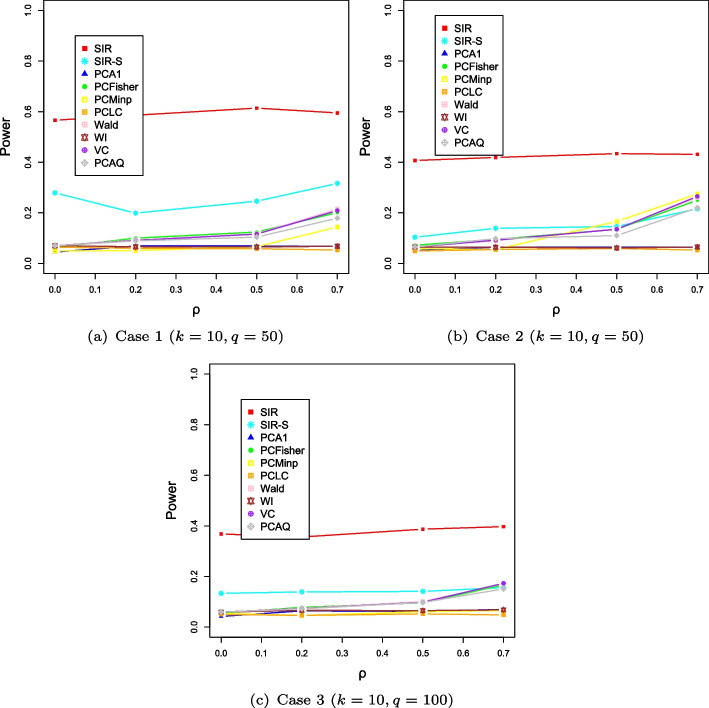
The evolution of power along with the varying correlation $\rho$ in Scenario 2

Simulation results for power comparisons are shown in Fig. 3. Figure 3 shows that the powers of our SIR and SIR-S methods decrease when the effect vectors are in mixed directions for the high-dimensional phenotypes. However, effect vectors in mixed directions do not affect the power of the PC-based methods. From these observations, we can see that our SIR and SIR-S methods are sensitive to the direction of effect vector for high-dimensional phenotypes. Clearly, the powers of all methods are affected by the dimensional increase of the phenotype to a certain degree. Compare to Scenario 1, the powers of both the SIR and SIR-S methods are somewhat decreased, but still, our methods outperform the competing methods in most cases.

### Scenario 3: high-dimensional phenotype and high-dimensional genotype

We conduct additional simulations to compare the performance of our proposed tests with existing PC-based methods for both high-dimensional phenotype and genotype. From Scenario 2, we know that when effect vectors are in mixed directions, the powers of our proposed methods decrease in a high-dimensional phenotype setting. For a fair comparison with the PC-based methods, in this simulation, we consider the effect vector of the third SNP $\varvec{\beta }_{3}$ on the first five phenotypes to be of mixed directions. Specifically, we consider high-dimensional phenotype and genotype with four cases: Case 1 is for $k=40$, $q=50$; Case 2 is for $k=40$, $q=100$; Case 3 is for $k=100$, $q=50$; Case 4 is for $k=100$, $q=100$. Table 5 shows the setting of different effect vectors. The datasets are generated similarly to Scenario 1, but here the number of SNPs is $k=40$ or 100.

**Table 5:** The setting of effect vectors in Scenario 3

Case 1	$k=40,q=50$	$\varvec{\beta }_{3}=c(1.10,-1.10,1.10,-1.10,1.10,0.00,0.00,\dots ,0.00)$
		$$\varvec{\beta }_{4}=c(0.00,0.02,0.00,0.00,\dots ,0.00)$$
Case 2	$k=40,q=100$	$\varvec{\beta }_{3}=c(1.10,-1.10,1.10,-1.10,1.10,0.00,0.00,\dots ,0.00)$
		$$\varvec{\beta }_{4}=c(0.00,0.02,0.00,0.00,\dots ,0.00)$$
Case 3	$k=100,q=50$	$\varvec{\beta }_{3}=c(1.10,-1.10,1.10,-1.10,1.10,0.00,0.00,\dots ,0.00)$
		$$\varvec{\beta }_{4}=c(0.00,0.02,0.00,0.00,\dots ,0.00)$$
Case 4	$k=100,q=100$	$\varvec{\beta }_{3}=c(1.10,-1.10,1.10,-1.10,1.10,0.00,0.00,\dots ,0.00)$
		$$\varvec{\beta }_{4}=c(0.00,0.02,0.00,0.00,\dots ,0.00)$$

$*$ The default value of other effect vectors ${\varvec{\beta }_j}$’s are ${\textbf{0}}$

**Table 6:** Empirical type I errors based on 100,000 replicates in Scenario 3

*k*	*q*	$\rho$	SIR	SIR-S	PCA1	PCFisher	PCMinp	PCLC	Wald	WI	VC	PCAQ
40	50	0.0	0.04997	0.05142	0.05063	0.05189	0.04991	0.05029	0.04945	0.04879	0.04857	0.04997
		0.2	0.04895	0.05149	0.05157	0.04994	0.04845	0.04772	0.05022	0.05015	0.05107	0.05541
		0.5	0.05007	0.05057	0.05036	0.04734	0.05048	0.04951	0.05005	0.05302	0.05235	0.05326
		0.7	0.04989	0.05503	0.05091	0.05196	0.04980	0.04979	0.05076	0.04780	0.04993	0.04892
40	100	0.0	0.05167	0.05277	0.05494	0.04556	0.04956	0.04860	0.04956	0.04859	0.04877	0.05196
		0.2	0.04783	0.05167	0.04799	0.05147	0.05064	0.04958	0.04991	0.04703	0.04995	0.04986
		0.5	0.05105	0.05198	0.04875	0.04889	0.04962	0.04938	0.04855	0.04974	0.04978	0.04884
		0.7	0.05263	0.05135	0.04964	0.04879	0.05083	0.05045	0.04953	0.04820	0.04946	0.05278
100	50	0.0	0.05246	0.05205	0.04870	0.04569	0.04862	0.04974	0.04675	0.04687	0.04788	0.04967
		0.2	0.05076	0.05130	0.04886	0.04568	0.04787	0.04987	0.04873	0.05044	0.04656	0.04968
		0.5	0.05042	0.05178	0.04978	0.04467	0.04874	0.04956	0.04479	0.04870	0.04873	0.04994
		0.7	0.05201	0.05161	0.04874	0.04676	0.04979	0.05213	0.04678	0.04835	0.04658	0.05016
100	100	0.0	0.04982	0.05216	0.05033	0.04984	0.04989	0.05225	0.04863	0.04782	0.04659	0.04893
		0.2	0.04953	0.05573	0.05105	0.04882	0.04963	0.05195	0.04986	0.04983	0.04674	0.04942
		0.5	0.05014	0.05436	0.05073	0.04836	0.05127	0.05285	0.04983	0.04976	0.04734	0.04971
		0.7	0.04947	0.05174	0.04994	0.04903	0.04957	0.05316	0.05158	0.04994	0.04605	0.04996

Table 6 summarizes the simulation results for type I error estimates. It clearly shows that all methods can retain the empirical type I errors very well at the significance level.

**Fig. 4:**
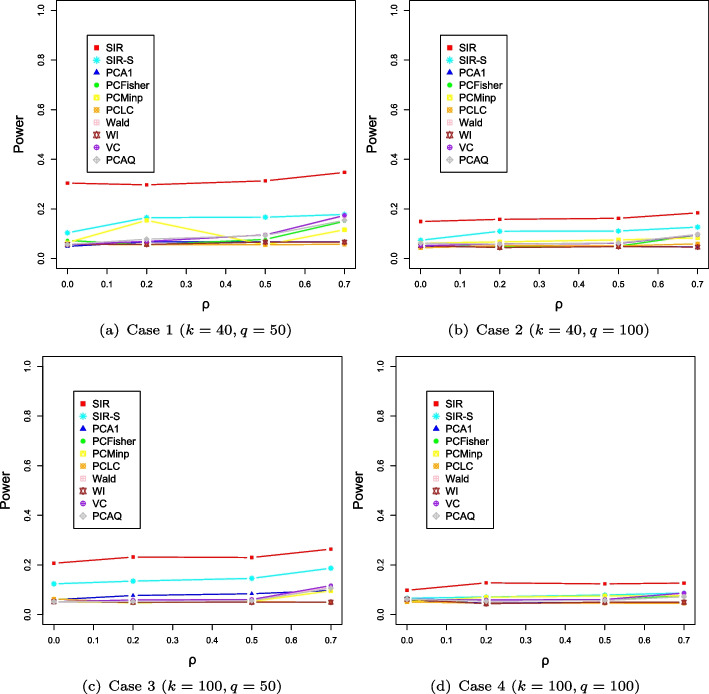
The evolution of power along with the varying correlation $\rho$ in Scenario 3

Figure 4 presents the simulation results of power comparisons for all settings. The powers of our SIR and SIR-S methods are reduced as the dimensions of both genes and phenotypes increase. Nevertheless, the powers of the proposed methods are still slightly higher than the PC-based methods.

### Scenario 4: simulation based on a real genotype data

In this section, we perform additional simulations to evaluate the performance of our SIR and SIR-S procedures on a more realistically simulated data, and compare with the other eight methods based on a real genotype data from the Genetic Analysis Workshop 17 (GAW17). The genotype data of 697 unrelated individuals are extracted from the sequence alignment files provided by the 1000 Genomes Project for their pilot3 study (http://www.1000genomes.org), in which we choose the TG gene as a candidate gene. The TG gene has 146 SNPs which encodes the thyroglobulin, one of the largest proteins in the human body, and mutation of the TG gene may cause hypothyroidism and autoimmune disorders [22].

In this simulation, the 100 dimensional phenotypes of the 697 individuals are generated from the model (1). To focus on the main points, six SNPs are selected as the causal variants. Specifically, the three SNPs, 20-th, 60-th, 100-th, are chosen to be far away and the others, 4-th, 6-th, 8-th, are chosen to be clustered. To consider the fact that the causal SNPs affect the disease in different directions, we set the effect vector of the each SNP $\varvec{\beta }_j$ on the first five phenotypes to be of mixed directions, while the rest of them are set to be ${\textbf{0}}$. We generate 100,000 simulated data sets for type I error evaluation and 1000 data sets for power comparison.

Table 7 lists the empirical type I errors of the ten methods of the association analysis for TG gene at the nominal level of 0.05. From Table 7, it is apparent to see that all the methods control the empirical type I errors of the TG gene very well. Table 8 shows the power comparison results of the ten methods for different settings. It clearly shows that all methods are robust to the proportion of the causal variants, and the SIR and SIR-S methods provide more power than the other methods in most cases.

**Table 7:** Empirical type I errors of the TG gene based on 100,000 replicates in Scenario 4

$\rho$	SIR	SIR-S	PCA1	PCFisher	PCMinp	PCLC	Wald	WI	VC	PCAQ
0.0	0.05001	0.05145	0.04510	0.04409	0.05040	0.05265	0.04551	0.04849	0.04776	0.05165
0.2	0.04998	0.05145	0.05017	0.04764	0.05044	0.04652	0.05135	0.05205	0.04657	0.05528
0.5	0.04985	0.05036	0.05648	0.04879	0.04966	0.05106	0.04667	0.05503	0.04986	0.05663
0.7	0.05113	0.04958	0.04989	0.04782	0.04661	0.05144	0.04876	0.05523	0.04975	0.05356

**Table 8:** Empirical powers of the TG gene based on 1000 replicates in Scenario 4

$\rho$	SIR	SIR-S	PCA1	PCFisher	PCMinp	PCLC	Wald	WI	VC	PCAQ
0.0	0.397	0.161	0.281	0.195	0.191	0.074	0.096	0.104	0.119	0.096
0.2	0.385	0.167	0.049	0.117	0.155	0.121	0.152	0.055	0.147	0.099
0.5	0.395	0.158	0.058	0.292	0.297	0.055	0.210	0.062	0.276	0.165
0.7	0.481	0.172	0.056	0.441	0.303	0.082	0.475	0.051	0.478	0.364

## Application to the sequencing data from ADNI

We analyze the ADNI1 and ADNI2 datasets from the Alzheimer’s Disease Neuroimaging Initiative (ADNI) study. The ADNI seeks to develop biomarkers of the disease and advance the understanding of AD (Alzheimer’s disease) pathophysiology, so as to improve diagnostic methods for early detection of AD and improve the clinical trial design. Additional goals are examining the rate of progress for both mild cognitive impairment and Alzheimer’s disease, as well as building a large repository of clinical and imaging data. ADNI is a study that assesses the effects of genetic variants on AD and various AD-related outcomes, including 3D brain imaging and cognitive measurements [23]. Proteolytic fragments of amyloid and post-translational modification of tau species in Cerebrospinal fluid (CSF) as well as cerebral amyloid deposition are important biomarkers for AD [24, 25].

A total of 800 subjects are included in the data, with 200 normal controls, 400 mild cognitive impairment (MCI), and 200 mild AD. We are interested in the association between genetic variants and five outcomes, including the hippocampus, entorhinal, amyloid beta (A$\beta _{42}$), tau, and phosphorylated tau (ptau_181_) levels. It has been reported that the AOPE gene is related to AD and its associated outcomes [26]. Therefore, as in [27], SNP rs769449 in gene AOPE is selected in our study. We also include 15 SNPs around rs769449 in our study: 8 SNPs on the left of rs769449 and 7 SNPs on the right, respectively. The SNPs rs8106922, rs1160985, and rs394819 are located in an intronic region of gene TOMM40, while other SNPs rs1081101, rs405509, and rs769449 are in the gene APOE, and rs445925 in gene APOC1. In the preprocessing step, we exclude the subjects which missing outcomes and genetic variants. After quality control, a total of 453 subjects are available in our study.

We conduct an association study to identify genetic factors influencing the five outcomes. All the aforementioned methods are performed with the nominal level of significance $\alpha =0.05$. Since the PC-based methods focus on the association test of a single marker, here we apply Bonferroni correction to adjust for multiple testing involving 16 markers ($\alpha _{\text {Bonferroni}}=0.05/16=0.0031$). Four SNPs are detected by SIR method, including kgp8001324 ($p=2.3\times 10^{-2}$), rs405509 ($p=5.3\times 10^{-3}$), rs769449 ($p=1.1\times 10^{-3}$), and rs445925 ($p=4.4\times 10^{-2}$). Among them, two SNPs rs405509 and rs769449 are in the gene APOE, and rs445925 in the gene APOC1. Note that APOE and TOMM40 are well-known genes associated with AD [28]. In particular, the SNPs kgp8001324, rs405509, and rs769449 are detected by our SIR method and other comparative methods, justifying the effectiveness of the proposed method. Meanwhile, the SNP rs445925 in gene APOC1 can be detected only by the SIR method, and APOC1 gene is reported to be a genetic risk factor for dementia and cognitive impairment in the elderly and it has a significant impact on hippocampal volumes [29]. The *p* value of PCLC for detecting SNP kgp8001324 ($p=7.31\times 10^{-5}$) is more significant than the SIR. As for SNP rs405509 in the gene APOE, the *p* value of PC5 is $3\times 10^{-3}$ similar to the SIR methods. The SNP rs769449 ($p=1.01\times 10^{-4}$) in the gene APOE is also detected by PC5.

**Table 9:** Comparison results of *p* value for detected SNPs by all methods using five traits

Method	Number of detected SNPs	SNP	*p* value
SIR	4	kgp8001324	2.30e−02
		rs405509	5.30e−03
		rs769449	1.10e−03
		rs445925	4.40e−02
PC1	1	rs 8106922	3.00e−03
PC5	4	rs394819	2.00e−03
		rs405509	3.00e−03
		rs769449	1.01e−04
		kgp21335103	3.00e−03
PCFisher	2	rs769449	5.99e−04
		kgp21335103	5.03e−04
PCLC	1	kgp8001324	7.31e−05
PCMin	1	kgp2187574	8.11e−04
VC	1	kgp8001324	2.00e−03
WI	1	kgp2187574	4.01e−04
Wald	1	kgp2187574	8.82e−04
PCAQ	1	rs769449	2.02e−04

The SNPs rs769449, rs405509, and kgp8001324 are detected by the SIR method as well as several comparative methods, which verifies the fact that these SNPs are associated with AD. In a nutshell, the SIR and PC5 methods, which detect four SNPs, perform better than other methods, but only the SIR method can detect one important SNP rs445925. In short, the SIR detects most SNPs across all cases, further confirming the advantages of the proposed method. We summarize a subset of the detected SNPs in Table 9.

## Discussion

With the rapid development of next-generation sequence technologies, millions of SNPs and outcomes are usually collected in recent GWAS, and the high dimensionality of data has become a great challenge to statistical analysis. Furthermore, considering the complex correlations between multiple traits will be beneficial in revealing more latent information. In contrast to univariate analysis, multivariate analysis can exploit the correlations among phenotypes to improve power, in which a flexible framework is strongly essential for testing the association between multiple predictors and multiple outcomes.

In this paper, we proposed a novel SIR-based association test that enables the analysis of multiple traits while taking into account the similarity between one or more traits to facilitate information borrowing. First, this procedure could preserve important information about the original regression between responses $\varvec{y}$ and predictors $\varvec{g}$ during carrying out the dimension reduction. To this end, we divided the range of $\varvec{g}$ according to genotype similarity and estimated the genetic relatedness matrix to measure genetic similarity between individuals during dimension reduction of phenotype $\varvec{y}$ for the proposed method. Then, we assigned the individuals with similar genotypes to the same group, followed by conducting reduction steps, which significantly improved the computing speed. Second, several scenarios with low- and high-dimensional responses and genotypes were considered in our simulations. Our numerical studies illustrate that the powers of the SIR and SIR-S methods decrease as the genotype dimension *k* increases in low-dimensional phenotypes setting, where the PC-based methods exhibit comparable performances to our proposed method. In the high-dimensional phenotypes setting, we found that the direction of the effect vector has mixed direction, and the powers of proposed methods were reduced but with little effect on the PC-based methods. Finally, we conducted real-data analysis with five outcomes. Among several methods, the important SNP rs445925 in gene APOC1, which has a significant impact on hippocampal volumes, was detected only by our SIR-based method. Unlike the other methods, the SIR-based method also detected most SNPs across all cases. The analysis of ADNI data has shown that the proposed method can reveal biologically meaningful genetic markers with reasonable prediction accuracy and stability, providing suggestions for further clinical or epidemiological research. Through real-data analysis, we further confirmed that our method is more conducive to understanding the underlying genetic architecture in the multiple phenotype studies.

Note that our method cannot be applied to GWAS data in that the model (7) is not suitable to it. Although we can test each SNP one by one based on the model (8) to perform GWAS by adjusting for multiple testing theoretically, the procedure of SIR-based dimension reduction of phenotype needs to merge adjacent slices based on the genetic relatedness matrix which is estimated through the empirical correlation between two individuals. Therefore, it becomes increasingly challenging to guarantee gene similarity when there are more SNPs.

## Conclusion

There are still some problems not be worked out, which will be investigated in our upcoming research. Here, we adopted the SIR-based method to estimate the central subspace, but other methods such as the sliced average variance estimation (SAVE) [30] and the directional regression (DR) [31] are also worth trying in the future. In addition, this paper only considered the case of one component $d_0 = 1$, but correlation analysis with multiple components can be similarly considered. We hope that the proposed methods can help in the search for genetic variants of complex diseases, and stimulate further interest and research in developing statistical methods for the analysis of next-generation sequence data.

## Data Availability

Data used in the preparation of this article were obtained from the Alzheimer’s Disease Neuroimaging Initiative (ADNI) database at https://adni.loni.usc.edu/data-samples/access-data but restrictions apply to the availability of these data, which were used under license for the current study, and so are not publicly available. The ADNI was launched in 2003 as a public-private partnership, led by Principal Investigator Michael W. Weiner,MD. The primary goal of ADNI has been to test whether serial magnetic resonance imaging (MRI), positron emission tomography (PET), other biological markers, and clinical and neuropsychological assessment can be combined to measure the progression of mild cognitive impairment (MCI) and early Alzheimer’s disease (AD).

## References

[1] Zhu W, Zhang H (2009). Why do we test multiple traits in genetic association studies?. J Korean Stati Soc.

[2] Liu Z, Lin X (2019). A geometric perspective on the power of principal component association tests in multiple phenotype studies. J Am Stat Assoc.

[3] Hilafu H, Safo SE, Haine L (2020). Sparse reduced-rank regression for integrating omics data. BMC Bioinform.

[4] Maity A, Sullivan PF, Tzeng J-I (2012). Multivariate phenotype association analysis by marker-set kernel machine regression. Genet Epidemiol.

[5] Broadaway KA, Cutler DJ, Duncan R, Moore JL, Ware EB, Jhun MA, Bielak LF, Zhao W, Smith JA, Peyser PA (2016). A statistical approach for testing cross-phenotype effects of rare variants. Am J Hum Genet.

[6] Maier R, Moser G, Chen G-B, Ripke S, Absher D, Agartz I, Akil H, Amin F, Andreassen OA, Anjorin A (2015). Joint analysis of psychiatric disorders increases accuracy of risk prediction for schizophrenia, bipolar disorder, and major depressive disorder. Am J Hum Genet.

[7] Lange C, Silverman EK, Xu X, Weiss ST, Laird NM (2003). A multivariate family-based association test using generalized estimating equations: FBAT-GEE. Biostatistics.

[8] Korte A, Vilhjálmsson BJ, Segura V, Platt A, Long Q, Nordborg M (2012). A mixed-model approach for genome-wide association studies of correlated traits in structured populations. Nat Genet.

[9] Chiu C-Y, Jung J, Wang Y, Weeks DE, Wilson AF, Bailey-Wilson JE, Amos CI, Mills JL, Boehnke M, Xiong M (2017). A comparison study of multivariate fixed models and gene association with multiple traits (gamut) for next-generation sequencing. Genet Epidemiol.

[10] Chiu C, Jung J, Chen W, Weeks DE, Ren H, Boehnke M, Amos CI, Liu A, Mills JL, Ting Lee M-L (2017). Meta-analysis of quantitative pleiotropic traits for next-generation sequencing with multivariate functional linear models. Eur J Hum Genet.

[11] Wang Y, Liu A, Mills JL, Boehnke M, Wilson AF, Bailey-Wilson JE, Xiong M, Wu CO, Fan R (2015). Pleiotropy analysis of quantitative traits at gene level by multivariate functional linear models. Genet Epidemiol.

[12] Zhang H, Liu C-T, Wang X (2010). An association test for multiple traits based on the generalized Kendall’s tau. J Am Stat Assoc.

[13] Zhu W, Jiang Y, Zhang H (2012). Nonparametric covariate-adjusted association tests based on the generalized Kendall’s tau. J Am Stat Assoc.

[14] Yang JJ, Li J, Williams L, Buu A (2016). An efficient genome-wide association test for multivariate phenotypes based on the fisher combination function. BMC Bioinform.

[15] Conneely KN, Boehnke M (2007). So many correlated tests, so little time! Rapid adjustment of $$p$$ values for multiple correlated tests. Am J Hum Genet.

[16] Sluis S, Posthuma D, Dolan CV (2013). TATES: efficient multivariate genotype-phenotype analysis for genome-wide association studies. PLoS Genet.

[17] Cook RD (1996). Graphics for regressions with a binary response. J Am Stat Assoc.

[18] Li K-C (1991). Sliced inverse regression for dimension reduction. J Am Stat Assoc.

[19] Cook RD (1998). Regression graphics: ideas for studying regressions through graphics.

[20] Huang M-Y, Hung H (2022). A review on sliced inverse regression, sufficient dimension reduction, and applications. Stat Sin.

[21] Thompson EA (2013). Identity by descent: variation in meiosis, across genomes, and in populations. Genetics.

[22] Mizuma T, Watanabe M, Inoue N, Arakawa Y, Tomari S, Hidaka Y, Iwatani Y (2017). Association of the polymorphisms in the gene encoding thyroglobulin with the development and prognosis of autoimmune thyroid disease. Autoimmunity.

[23] Saykin AJ, Shen L, Foroud TM, Potkin SG, Swaminathan S, Kim S, Risacher SL, Nho K, Huentelman MJ, Craig DW (2010). Alzheimer’s disease neuroimaging initiative biomarkers as quantitative phenotypes: genetics core aims, progress, and plans. Alzheimer’s Dement.

[24] Li QS, Parrado AR, Samtani MN, Narayan VA, Initiative ADN (2015). Variations in the FRA10AC1 fragile site and 15q21 are associated with cerebrospinal fluid a$$\beta $$1-42 level. PLoS ONE.

[25] Kim S, Park S, Chang I (2022). Development of quantitative and continuous measure for severity degree of Alzheimer’s disease evaluated from MRI images of 761 human brains. BMC Bioinform.

[26] Hoffmann K, Sobol NA, Frederiksen KS, Beyer N, Vogel A, Vestergaard K, Brændgaard H, Gottrup H, Lolk A, Wermuth L (2016). Moderate-to-high intensity physical exercise in patients with Alzheimer’s disease: a randomized controlled trial. J Alzheimers Dis.

[27] Deming Y, Li Z, Kapoor M, Harari O, Del-Aguila JL, Black K, Carrell D, Cai Y, Fernandez MV, Budde J (2017). Genome-wide association study identifies four novel loci associated with Alzheimer’s endophenotypes and disease modifiers. Acta Neuropathol.

[28] Maruszak A, Pepłońska B, Safranow K, Chodakowska-Żebrowska M, Barcikowska M, Żekanowski C (2012). TOMM40 rs10524523 polymorphism’s role in late-onset Alzheimer’s disease and in longevity. J Alzheimers Dis.

[29] Serra-Grabulosa J, Salgado-Pineda P, Junque C, Sole-Padulles C, Moral P, Lopez-Alomar A, Lopez T, Lopez-Guillen A, Bargallo N, Mercader J (2003). Apolipoproteins E and C1 and brain morphology in memory impaired elders. Neurogenetics.

[30] Cook RD, Weisberg S (1991). Discussion of sliced inverse regression for dimension reduction. J Am Stat Assoc.

[31] Li B, Wang S (2007). On directional regression for dimension reduction. J Am Stat Assoc.

